# Bidirectional Relationship Between Cancer and Heart Failure: Insights on Circulating Biomarkers

**DOI:** 10.3389/fcvm.2022.936654

**Published:** 2022-07-06

**Authors:** Michela Chianca, Giorgia Panichella, Iacopo Fabiani, Alberto Giannoni, Serena L'Abbate, Alberto Aimo, Annamaria Del Franco, Giuseppe Vergaro, Chrysanthos Grigoratos, Vincenzo Castiglione, Carlo Maria Cipolla, Antonella Fedele, Claudio Passino, Michele Emdin, Daniela Maria Cardinale

**Affiliations:** ^1^Institute of Life Sciences, Scuola Superiore Sant'Anna, Pisa, Italy; ^2^Cardiology Division, Fondazione Toscana Gabriele Monasterio, Pisa, Italy; ^3^Cardiology Division, Pisa University Hospital, Pisa, Italy; ^4^Cardioncology Unit, Cardioncology and Second Opinion Division, European Institute of Oncology, Istituto di Ricovero e Cura a Carattere Scientifico (I.R.C.C.S.), Milan, Italy

**Keywords:** cancer, inflammation, cardiovascular disease, cardio-oncology, circulating biomarkers, neuro-hormonal activation

## Abstract

Cancer and heart failure are the two leading causes of death in developed countries. These two apparently distinct clinical entities share similar risk factors, symptoms, and pathophysiological mechanisms (inflammation, metabolic disturbances, neuro-hormonal and immune system activation, and endothelial dysfunction). Beyond the well-known cardiotoxic effects of oncological therapies, cancer and heart failure are thought to be tied by a bidirectional relationship, where one disease favors the other and vice versa. In this context, biomarkers represent a simple, reproducible, sensitive and cost-effective method to explore such relationship. In this review, we recapitulate the evidence on cardiovascular and oncological biomarkers in the field of cardioncology, focusing on their role in treatment-naïve cancer patients. Cardioncological biomarkers are useful tools in risk stratification, early detection of cardiotoxicity, follow-up, and prognostic assessment. Intriguingly, these biomarkers might contribute to better understand the common pathophysiology of cancer and heart failure, thus allowing the implementation of preventive and treatment strategies in cardioncological patients

## Introduction

Cardiovascular disease, namely heart failure (HF), is the leading cause of death in industrialized countries, and its incidence is increasing. Recently, several studies have drawn the scientific community attention to the overlap between these two diseases, which were previously considered as distinct from each other (1). In particular, the bidirectional relationship between cancer and HF has been highlighted, demonstrating the presence of common risk factors (such as aging, male sex, obesity, diabetes mellitus, sedentariness and smoking) as well as shared etiopathogenetic pathways in both diseases (2). Oxidative stress and inflammation have been implicated in the pathogenesis of cancer and cardiovascular diseases, promoting the tumor microenvironment and cancer invasiveness on the one hand, and inducing endothelial dysfunction, fibrotic processes, and the formation of atherosclerotic plaques on the other (3). The renin-angiotensin-aldosterone system (RAAS) has also been shown to play a role in certain steps of cancer development, along with typical biomarkers of cardiac stress (3). In contrast, some preclinical studies have suggested a possible role for some oncometabolites (e.g., D-2-hydroxyglutarate) in promoting HF (4). As a result of this evidence, in recent years there has been a growing interest in the new field of cardioncology, aimed at understanding and analyzing the precise biological mechanisms underlying the overlap between these two diseases.

**Figure 1:**
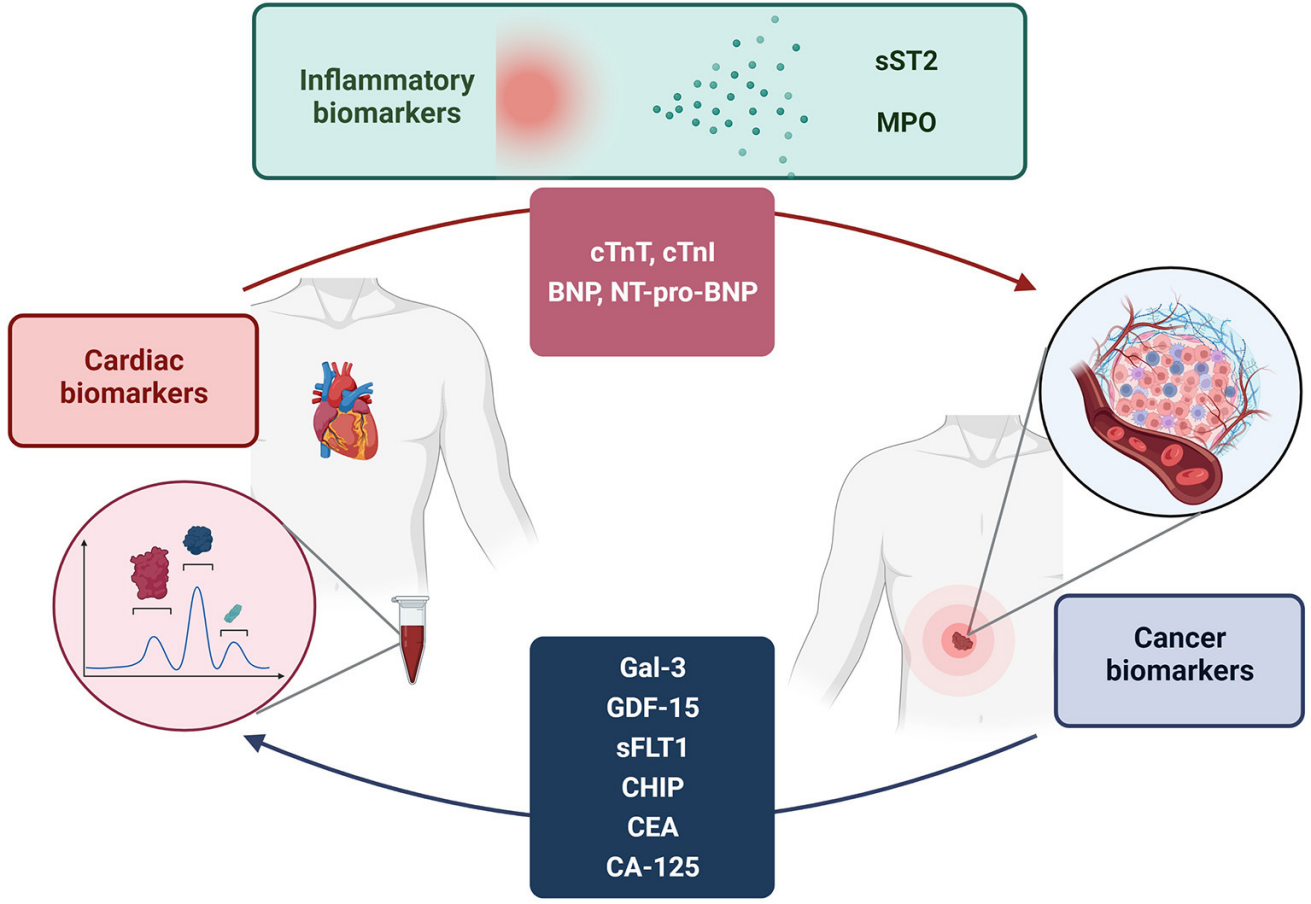
Bi-directional relationship between cancer and cardiovascular disease. Analysis of the elevation of disease-specific biomarkers in both conditions suggests a possible influence of cancer on the pathogenesis of cardiovascular disease and vice versa. The elevation of biomarkers is also justified by several pathogenetic pathways shared by both diseases.

In this narrative review, we provide an overview of the main biomarkers of cardiovascular disease and cancer, their pathophysiological role and their overlap in the two diseases (Figure 1). The proper use of these biomarkers for diagnostic and therapeutic purposes is still unclear, and future studies will be needed to eventually introduce them into clinical practice.

## The Role of Cardiovascular Biomarkers in Cancer

Cardiac biomarkers have been investigated as inexpensive and easily accessible tools for risk assessment, prediction of response to treatment, early diagnosis of cardiotoxicity, as well as monitoring disease progression and evaluating the prognosis of cancer-related cardiac involvement (5). However, while there is extensive literature on the role of cardiac biomarkers for the early detection of cardiotoxicity from cancer therapies (6, 7), much less is known about cardiac biomarkers role before the start of chemotherapy (8). One of the best settings to explore the bidirectional relationship between cancer and HF is, indeed, the one considering treatment-naïve cancer patients (8) [Table 1, (9–14)]. In such case, an elevation of cardiovascular biomarkers may result from cardiovascular comorbidities, systemic perturbances (e.g., inflammation, oxidative stress, sepsis) or, more intriguingly, as a direct effect of cancer itself.

**Table 1:** Prognostic value of cardiovascular biomarkers in treatment-naïve cancer patients.

**Biomarker**	**References**	**Cut-off/ range of value found**	**Cancer type**	**Association with cancer outcome**
cTnT	Pavo et al. (9)	≥0,005 ng/mL	Several types	Increased mortality risk (HR 1.21, *p* <0.001)
cTnT	Kitayama et al. (10)	Not defined	Breast	No predictive value of cardiotoxicity
cTnT	Zardavas et al. (11)	>14 ng/L	Breast	Increased cardiotoxicity risk (HR 3.57, *p* < 0.001)
cTnI	Zardavas et al. (11)	>40 ng/L	Breast	Increased cardiotoxicity risk (HR 4.52, *p* < 0.001)
cTnT	Petricciuolo et al. (12)	≥14 ng/L	Lung	TnT predicted CV death, stroke or TIA, pulmonary embolism and new-onset HF
cTnT	Rini et al. (13)	Not defined	Advanced renal cell carcinoma	Increased risk of MACE (RR 3.31)
NT-proBNP	Pavo et al. (9)	≥125 pg/mL	Several types	Increased mortality risk (HR 1.54, *p* < 0.001)
NT-proBNP	Rini et al. (13)	Not defined	Advanced renal cell carcinoma	No increased risk of MACE
BNP	Rini et al. (13)	Not defined	Advanced renal cell carcinoma	No increased risk of MACE
Neprilysin	Pavo et al. (14)	Median values 276 pg/ml	Several types	Lack of association with mortality but for myelodysplastic disease (HR 1.27, *p* = 0.044)

### Cardiac Troponins

Cardiac troponin T and I (cTnT and cTnI, respectively) are cardiac regulatory proteins that control the calcium-mediated interaction between actin and myosin in cardiomyocytes (15). The troponin complex consists of three subunits: TnT, which binds to tropomyosin and facilitates contraction; TnI, which binds to actin and inhibits actin-myosin interactions; and troponin C, which binds to calcium ions (16). The majority of cTns is bound to myofilaments, and the remainder is free in the cytosol, which accounts for 3%−8% of the total amount (17). After disruption of the sarcolemmal membrane of the cardiomyocyte, troponin from the cytoplasmic pool is initially released, followed by a more protracted release from myofibril-bound cytosolic complexes (18, 19). However, an increase in serum troponin levels may rely not only on direct myocardial damage, but also demand ischemia, myocardial ischemia, myocardial strain due to volume and pressure overload, and chronic kidney disease (CKD) (16). Methods for determining cTnT and cTnI, first developed in the late 90s, have been continuously improved, increasing analytical sensitivity and specificity (20). Nowadays, highly sensitive (hs) immunoassays are available to determine hs-cTnT and hs-cTnI concentrations; they allow detecting very low but diagnostically significant concentrations of cardiac troponins in blood serum (17).

Cardiac troponins are the gold standard biomarkers for the detection of myocardial injury, namely in the setting of acute coronary syndromes (ACS) (21). In peripheral blood, troponins begin to rise within 3–4 h after the onset of myocardial injury and remains increased for 10–14 days (22). However, increased levels may be found in several different conditions, both physiological (e.g., physical exertion or psycho-emotional stress) and pathological, including chronic HF, diabetes, arterial hypertension, inflammatory heart disease, pulmonary embolism, chronic renal failure and sepsis (20, 21, 23).

Cardiac troponins are also the most widely used biomarkers to detect cardiotoxicity in cardioncology (24). With the advent of hs assays, it is possible to detect early subclinical cardiomyocyte damage and help provide treatments to prevent cardiotoxicity prior to the development of irreversible left ventricular (LV) dysfunction (24). In addition to this, cardiac troponins have been increasingly studied in cancer patients before receiving oncological treatment: cTnI was found to be significantly higher in 25 anthracycline-naïve cancer patients (36.5 pg/ml; 95% confidence interval [CI], 25.1–47.9 pg/ml), as compared to 60 healthy controls (*p* < 0.01) (25), and in 25 patients with ovarian cancer prior to treatment, as compared to women with endometriosis or benign ovary masses (26). A study on 452 treatment-naïve women with breast cancer explored the prognostic value of troponins I, T, and N-terminal prohormone of brain natriuretic peptide (NT-proBNP) to predict baseline susceptibility to trastuzumab-related cardiac dysfunction (11). Elevated baseline troponin I (>40 ng/L) and T (>14 ng/L), occurring in 56 of 412 (13.6%) and 101 of 407 (24.8%) patients, respectively, were associated with an increased significant LV ejection fraction (EF) drop risk (hazard ratio [HR] 4.52, *p* < 0.001 and HR 3.57, *p* < 0.001, respectively). A similar conclusion for NT-proBNP could not be drawn because of the lack of a well-established elevation threshold; however, higher increases from baseline were seen in patients with cardiotoxicity compared with patients without (11).

The prognostic role of hs-TnT before immune checkpoint inhibitors (ICIs) treatment start has been investigated for the first time in 30 patients with different lung cancer types (12). The primary endpoints considered in the study were cardiovascular death, stroke or transient ischemic attack, pulmonary embolism and new-onset HF, while the secondary endpoint was progression of cardiac involvement. After 3 months of follow-up from the ICIs treatment start, 14 ng/L proved to be the best cutoff for both primary (100% sensitivity, 73% specificity) and secondary endpoints (sensitivity 75%, specificity 77%). The primary endpoint occurred only in patients with hs-TnT ≥14 ng/L at baseline (12). Data from the phase III JAVELIN Renal 101 trial have also recently shown that patients with advanced renal cell carcinoma and higher baseline TnT are at increased risk of developing major adverse cardiovascular events (MACE) after receiving a combined treatment with ICIs and vascular endothelial growth factor receptor (VEGFR) inhibitors (relative risk [RR] 3.31; 95% CI, 1.19–9.22) (13). Other cardiac biomarkers (TnI, natriuretic peptides [NPs], and creatine kinase MB) measured at baseline were not significantly predictive for MACE (13).

### Natriuretic Peptides

NPs are a family of structurally related peptide hormones mainly produced by cardiovascular, brain and renal tissues (27, 28). Atrial natriuretic peptide (ANP) is a 28-amino acid peptide that is synthesized, stored, and released by atrial myocytes mainly in response to cardiomyocytes mechanical stretch due to volume overload (29). Other factors leading to ANP secretion include exercise, hypoxia and cold, as well as angiotensin, endothelin, vasopressin, catecholamine and glucocorticoid stimulation (30). Brain-type natriuretic peptide (BNP) is a 32-amino acid peptide that is largely synthesized by the ventricles and the brain, where it was first identified (31). BNP is first synthesized as pre-pro-BNP, which is then cleaved to pro-BNP, which proteolysis by furin (or corin) results in the active BNP and the inactive NT-pro-BNP (76 amino acids) (32). Various causes induce BNP synthesis in cardiomyocytes, such as tissue hypoxia, transmural pressure or volume overload, and pro-inflammatory cell factors (e.g., interleukin-1β [IL-1β], interleukin-6 [IL-6] and tumor necrosis factor-α [TNF-α] (30, 33).

NPs mediate a wide range of physiologic effects achieved by interaction with specific guanylyl cyclase receptors (30), including direct vasodilation of veins and arteries, respectively lowering central venous pressure (i.e., preload) and systemic vascular resistance and arterial pressure (i.e., afterload) (34). In the kidney, NPs induce natriuresis and diuresis, suppress renin secretion and aldosterone synthesis, and increase glomerular filtration rate by vasodilating afferent arterioles (35). In addition, NPs provide antiproliferative, antihypertrophic, and antifibrotic effects, thus hindering adverse cardiac remodeling (36, 37).

Both BNP and NT-proBNP are useful biomarkers routinely used in the diagnosis, risk stratification, therapy management and prognosis assessment in patients with acute or chronic HF (38). Increased levels of NPs are also found in the setting of pulmonary diseases, cardiac inflammatory or infectious diseases, endocrine disorders and high output status, such as sepsis, kidney failure, liver cirrhosis, and intracranial pathologies (39).

NPs have been reported to be markedly and constantly increased in the cancer population. NPs typically increase after treatment with various oncological treatments, namely anthracyclines (40). However, NPs elevation may also depend on causes other than cancer therapy, such as release from cancer cells themselves (41), volume overload (42) or cancer-related systemic inflammation (43). Burjonroppa et al. first demonstrated that there is lack of association between markedly elevated BNP levels (>1,000 pg/mL) and clinical evidence of volume overload or LV dysfunction in cancer patients with multiple comorbidities (44). On the contrary, Popat et al. found that very high NT-proBNP (>3,000 pg/mL) in cancer patients is usually encountered in the context of fluid overload and most often in hematologic malignancies (42). In a study by Sachiko et al., both plasma BNP and serum C-reactive protein (CRP) levels were significantly higher in cancer patients before treatment than non-cancer patients (43). There was also a significant positive correlation between plasma BNP and serum CRP levels in cancer patients (R = 0.360, *p* < 0.01) but not in those without. In cancer patients, CRP correlated with BNP independent of the age, creatinine level, hypertension, and body mass index (43).

In a prospective cohort study, Pavo et al. enrolled 555 patients with a primary diagnosis of cancer and no prior oncological therapies. NT-proBNP, mid-regional pro-atrial natriuretic peptide (MR-proANP), mid-regional pro-adrenomedullin (MR-proADM), C-terminal pro-endothelin-1, copeptin, hsTnT, proinflammatory markers IL-6 and CRP, cytokines serum amyloid A, haptoglobin and fibronectin have been measured (9). All cardiovascular hormones and hsTnT levels rose with tumor stage progression. All markers were significant predictors of mortality with HRs of 1.54 (95% CI; 1.24–1.90, *p* < 0.001) for NT-proBNP, and 1.21 (95% CI; 1.13–1.32, *p* < 0.001) for hsTnT, independent of age, gender, tumor entity and stage, and presence of cardiac comorbidities. NT-proBNP, MR-proANP, MR-proADM and hsTnT displayed a significant correlation with the inflammatory markers IL-6 and CRP. This study showed for the first time that cardiovascular hormones are related to cancer disease progression and severity, suggesting the presence of subclinical functional and morphological myocardial damage independent of cancer treatment (9). Thereafter, the authors tried to assess the prognostic role of neprilysin, an enzyme degrading NPs, in the same population. Although neprilysin seems to be involved in tumor biology as well as in cardiovascular diseases, no association was observed between neprilysin levels and overall survival (*p* = 0.887) except for myelodysplastic malignancies (HR 1.27; 95% CI, 1.01–1.61; *p* = 0.044) (14).

To summarize, cardiovascular biomarkers (cardiac troponins and NPs) are found to be remarkably increased in the cancer population. This may depend on either a direct or indirect cancer-related systemic perturbance, which is also fostered by aging-linked phenomena of inflammation and oxidative stress. Further studies are therefore needed to define the precise role of these biomarkers in clinical practice in terms of risk prediction, screening, and therapeutic monitoring of both HF and cancer.

## The Role of Oncological Biomarkers in Cardiovascular Disease

Cancer is a complex disease marked by the uncontrollable proliferation of genetically abnormal cells, and it has long been the main cause of mortality in several countries (45). Early diagnosis, achieved through the use of specific biomarkers, is critical in terms of successful and timely therapy and patient survival. According to the National Institutes of Health, a biomarker is a feature that is objectively tested and assessed as a sign of normal biologic processes, pathogenic processes, or pharmaceutical reactions to a therapeutic intervention (46).

A reliable and appropriate biomarker must have several characteristics: it must be diagnostic while also allowing for early diagnosis, provide prognostic information, and have predictive potential to confirm therapy effectiveness (47). Enzymes, metabolites, DNA and RNA, as well as surface receptors, are all examples of cancer biomarkers (48). Several cancer biomarkers, such as the prostate-specific antigen and carcinoembryonic antigen (CEA), are now widely used in clinical practice and are universally regarded as useful diagnostic and prognostic tools (45). In recent years, studies investigating novel biomarkers in cancer have focused on the junction of malignancy and cardiovascular disease at several levels (49) [Table 2, (6, 51–77)]; these findings could be linked to the already well-known cardiotoxicity of neoplastic agents and radiation therapy, as well as to the multiple common biological mechanisms in cancer and cardiovascular disease development (6, 77). Furthermore, multiple studies have found that patients with both cardiovascular disease and cancer had a greater mortality rate than patients with either condition alone, underscoring the importance of treating both diseases jointly (78). It is therefore essential to identify and study biomarkers shared by both diseases, which could be useful tools in diagnostic and prognostic terms, allowing to understand the complicated dialogue between these two conditions.

**Table 2:** Elevated cancer biomarkers in cardiovascular disease.

**Biomarker**	**References**	**Cut-off/ range of value found**	**Population characteristics**	**Association with CV disease**
CA-125	Toshihiko et al. (100)	>35 U/mL	HF Pericardial, metastasis, renal failure, HypothyroidismRF, hypothyroidism	Pericardial effusion
CA-125	Nägele et al. (50)	>35 U/mL	Patient with HF admitted for HTX, patients after HTX	Association with NPs, severity of HF, response to medical therapy
CA-125	D'aloia et al. (51)	>35 U/mL 68 ± 83 U/ml	CHF	CHF severity and short-term prognosis
CA-125	Turk et al. (52)	>35 U/mL 100.0 ± 129.4 U/ml	CHF	Pleural effusion
CA-125	Faggiano et al. (53)	NYHA classes III (60 ± 22 UI/ml) and IV (192 ± 115 UI/ml)	CHF	Severity of HF, response to medical therapy
CA-125	Durak-Nalbantic et al. (54)	71.05 [30.70–141.47] U/ml	CHF	Pleural effusion, pericardial effusion, decompensated HF
				
CEA	Faggiano et al. (53)	>5 ng/ml	CHF	No association with HF
CEA	Shi et al. (55)	>5 ng/ml	Patients of the BIOSTAT-CHF cohort	Association with NPs, prediction of all-cause mortality
CEA	Bracun et al. (56)	>5 ng/ml	UAE >10 mg/L	CV morbidity, CV mortality and all-cause mortality
				
Gal-3	Motiwala et al. (57)	>20 ng/ml	HF	Incidence **o**f CV events
Gal-3	Meijers et al. (75)	>17.8 ng/mL	HF	Risk of rehospitalization at 30, 60, 90, 120 days
Gal-3	Xi Zhang et.al (58)	>384,7 ng/mL*>9,76 ng/mL	HF	Diagnosis of HF
Gal-3	Veli Polat et al. (59)	>1,79 ng/mL	HFpEF	Diagnosis and severity of HFpEF
Gal-3	Medvedeva et al. (74)	>21 ng/mL	HF	Independent factor of death, correlation with oxidative stress and renal failure
GDF-15	Kempf et al. (60)	Δ 1,194–3,577 ng/L	HFrEF	All-cause mortality
GDF-15	Kempf et al. (61)	Δ 850–1,553 ng/L	Stable angina pectoris	Coronary heart disease mortality
GDF-15	Wang et al. (62)	Δ 306–14,493 ng/L	3,428 individuals from the Framingham Offspring Study	Death, HF, MACE
GDF-15	Schopfer et al. (63)	Δ 1,589–3,057 ng/L	CAD	All-cause mortality, CV events, MI, HF, hospitalization
GDF-15	Chan et al. (64)	Δ 1,555–4,030 ng/L Δ 1,812–4,176 ng/L	HFrEF; HFpEF	Death or HF hospitalization
GDF-15	Skau et al. (65)	None	AMI	Long-term predictor of all causes of mortality
PlGF and sFlt-1	Lenderink et al. (66)	> 27 ng/l	ACS	Adverse long-term outcomes
PlGF and sFlt-1	Hochholzer (2010)	>20 ng/L; >84 ng/L (sFlt-1)	Suspected MI	Mortality
PlGF and sFlt-1	Marković et al. (67)	>13.2 ng/L	NSTEMI	Short term death, decrease in renal function
PlGF and sFlt-1	Glaser et al. (68)	>19.5 ng/L	Suspected ACS	Risk of MACE
PlGF and sFlt-1	Matsui et al. (69)	>19.6 pg/mL	CKD	CV events, all-cause mortality
CHIP	Genovese et al. (70)	/	12,380 persons, unselected for cancer or hematologic phenotypes	Risk of CV disease
CHIP	Jaiswal et al. (71)	/	17,182 persons who were unselected for hematologic phenotypes	All-cause mortality, risks of incident coronary heart disease
CHIP	Calvillo et al. (72)	/	AML	Increased prevalence of CV diseases

*ACS, acute coronary syndrome; AMI, acute myocardial infarction; AML, acute myeloid leukemia; CA125, carbohydrate antigen 125; CAD, coronary artery disease; CEA, carcinoembryonic antigen; CHF, chronic heart failure; CHIP, clonal hematopoiesis of undetermined potential; CKD, chronic kidney disease; CV, cardiovascular; Gal-3, galectin-3; GDF-15, growth differentiation factor-15; HF, heart failure; HFpEF, heart failure with preserved ejection fraction; HFrEF, heart failure with reduced ejection fraction; HTX, heart Transplantation; MACE, major adverse cardiovascular events; MI, myocardial infarction; NP, natriuretic peptide; NSTEMI, non ST-segment elevation myocardial infarction; NYHA, new york heart association; PlGF, placental growth factor; sFlt-1, soluble fms-like tyrosine kinase-1; UAE, urinary album excretion*.

### CA125 and CEA

Serum carbohydrate antigen 125 (CA125), a high-molecular-weight membrane glycoprotein, is a peptide repeat epitope of the mucin MUC16. It has a C-terminal portion with a short cytoplasmic tail and a transmembrane domain, as well as an extracellular N-terminal region with numerous partially conserved tandem repeats (79). CA125 is the most well-studied serologic tumor marker used to assess the clinical status of ovarian cancer patients and for the differential diagnosis of pelvic masses (80, 81).

CA125 antigen is not exclusively expressed on ovarian-cancer tumor cells. Acute leukemia, non-lymphoma Hodgkin's, melanoma, breast and lung cancers, and gastrointestinal carcinoma have all been linked to increased serum CA125 levels (82). Furthermore, CA125 expression has also been found under physiological conditions in tissues of mesothelial origin, such as the pleura, pericardium, and peritoneum (83). Moreover, in subsequent research, it has also been detected in the kidney, gallbladder, pancreas, lung, stomach, and colon, implying that CA125 lacks organ specificity due to its extensive diffusion across the body tissues (84, 85).

The clinical usefulness of the CA125 assay has been investigated in preclinical, translational, and clinical research since the 1980s (86–88). Firstly, in a cohort of 101 patients with ovarian cancer, Bast et al. found a positive CA125 antigen in 82% of patients, using a cutoff of 30–35 U/ml (89). Moreover, in subsequent research, the level of tumor biomarker elevation was found to be dependent on factors such as the type of tumor histology and the stage of the disease (84, 85).

In addition to its widespread usage as a predictive and diagnostic biomarker for ovarian cancer and other malignancies, CA125 has been shown to have a possible role in cardiovascular disease. Specifically, several clinical studies have found a correlation between elevation of tumor biomarker, pericardial effusion (73), and LV dysfunction (50).

In a cohort of patients with chronic HF, D'Aloia et al. found that elevated CA125 levels were associated to the severity of the New York Heart Association (NYHA) class. Furthermore, CA125 levels were also shown to be lower in patients with moderate to severe chronic HF who were getting aggressive pharmacological therapy, suggesting that the biomarker could be used to verify the treatment's therapeutic efficacy. Additionally, in short-term follow-up, CA125 has also been found to be a valuable prognostic factor (51).

The pathophysiological mechanism causing the simultaneous rise of CA125 and the development of cardiovascular disease is still unknown: according to some studies, an increase in the tumor marker is linked to the inflammatory process that underpins cardiovascular disease, which is exacerbated by a change in the hydrostatic balance, resulting in the development of HF (90).

Similar to CA125, CEA, a glycoprotein overexpressed on the cell surface in the majority of colorectal cancer [CRC] patients, has shown to be altered in cardiovascular disease as well. CEA is an oncofetal antigen that was first discovered as a CRC marker in 1965 (91). It is produced physiologically in the fetus but it is found only in small amounts in normal adult cells (92). CEA is one of the most commonly overexpressed cell surface molecules in CRC, and it is responsible for activating the cytokine cascade via direct interaction with monocytes, which is essential for cell adhesion and metastatic spread of intestinal cancer (93). However, CEA appears to be an insufficiently sensitive tool for primary CRC screening, especially in the early stages of the disease (sensitivity and specificity for Dukes' A and B 36 and 87%, respectively) (94).

Although most patients do not show elevated CEA at the time of diagnosis, CEA levels and disease stage have been found to correlate, making CEA a significant prognostic predictor independent of histologic grade and Dukes' stage (95).

An association between CEA and chronic inflammation, one of the key promoters of the development of various cardiovascular diseases including atherosclerosis, myocardial infarction, and HF (96, 97), has been proposed due to the strong correlation between CEA and elevated leukocyte counts (98).

In a study involving 2,079 patients from the BIOSTAT-CHF cohort, Shi et al. (55) explored the relationship between several tumor biomarkers (including CEA) and the outcome of HF. CEA levels increased in lockstep with NT-proBNP across a 21-month follow-up period, and were associated with all-cause death (HR 1.45, 95% CI, 1.30–1.61; *p* = 0.0001) and cardiovascular mortality (HR 1.18, 95% CI, 1.06–1.32; *p* = 0.003). Similarly, Bracun et al. (56) discovered a substantial connection between CEA levels and cardiovascular mortality over an 11.5-year follow-up period. CEA was also found to be associated with all-cause mortality and to be an independent predictor of cardiovascular events (56).

### Galectin-3

Human galectin-3 (Gal-3) is a 35-kDa protein belonging to the galectins family of galactoside-binding proteins. Its structure is characterized by the presence of a unique N-terminal domain followed by the carbohydrate recognition domain (CRD) (99). The first 12 amino acids of the protein are essential for its secretion or translocation from the cytoplasm to nucleus, whereas the CRD is crucial for binding glycoconjugates containing N-acetyllactosamine (99). Thus, although located mainly in the cytoplasm, Gal-3 can be found both inside and outside the cell, in the nucleus and on cell surface, as well as in biological fluids. Gal-3 is expressed in a variety of human tissues, including endothelium, epithelial cells, sensory neurons, and immune cells (101).

Gal-3 is involved in a variety of biological processes, and its roles vary depending on whether it is found inside or outside the cell; in the cytoplasm, it plays a key role in cell survival by inhibiting apoptosis through binding to Bcl-2 and influencing Ras-mediated AKT signaling (102). Nuclear-localized Gal-3, on the other hand, contributes to the formation of the spliceosome structure for pre-mRNA splicing and regulates gene transcription by enhancing the binding of specific transcription factors to the gene promoter. Finally, it appears to participate in cell-cell interactions in the extracellular environment, regulating cell adhesion and migration. Thus, Gal-3 plays a key role in several physiological processes including cell adhesion, angiogenesis, proliferation, inflammation and fibrosis (102).

Gal-3 has been linked to cancer in several studies, with evidence that it promotes neoplastic transformation, cell cycle progression, and apoptosis (103). According to Song et al., increased expression of Gal-3 was found in a K-ras mutant mouse model and in pancreatic cancer patients, and greater expression of Gal-3 was linked to increased tumor proliferation and infiltration (103). Furthermore, short hairpin RNA-induced downregulation of Gal-3 was demonstrated to reduce *in vitro* and *in vivo* tumor proliferation, invasion, and growth (103). Increased Gal-3 production improves cancer cell adherence to the extracellular matrix (ECM) which enhances, coupled with immune surveillance evasion, malignant cell movement and metastasis (104). According to preclinical research, increased serum levels of Gal-3 have been linked to a higher frequency of metastases (104). Furthermore, increased nuclear Gal-3 concentration promotes the expression of cyclin D1, thyroid transcription factor-1, and mucin 2, all of which are directly associated to cancer pathogenesis and progression (105). Gal-3 has also been discovered to have an intricate prognostic role, which changes according on the type of tumor: higher Gal-3 expression has been linked to a worse prognosis in numerous malignancies, including lymphoma, thyroid cancer, and leukemia, whereas lower Gal-3 expression has been linked to a worse outcome in prostate cancer and chronic lymphoblastic leukemia (106). The inconsistent results regarding Gal-3 expression based on tumor type could be related to differences in Gal-3 localization inside tumor cells, since the molecule's function differs depending on whether it is found in the nucleus, cytoplasm, or extracellular regions. Califice et al. have shown that nuclear Gal-3 has a pro-apoptotic effect in prostate cancer cells, whereas cytoplasmic Gal-3 has an anti-apoptotic effect (107). Although possible between Gal-3 and apoptosis-associated protein Nucling have been suggested, the exact pro-apoptotic pathway remains unknown (107).

Due to its proliferative action, Gal-3 has been intensively examined for a possible pathogenic role in cardiovascular disease and has been identified as a marker of fibrosis and inflammation. Specifically, Gal-3 appears to be involved in the development of HF and may act as a diagnostic and prognostic biomarker, suggesting higher rates of mortality and morbidity (58, 59). In a prospective cohort study, Medvedeva et al. found an increase in Gal-3 levels in patients with chronic HF of all NYHA classes. Gal-3 levels >21 ng/mL were also found to be an independent predictor of death across a 26-month follow-up, and were correlated to markers of oxidative stress, renal failure, and inflammation (74). Furthermore, Meijers et al. reported that plasma Gal-3 levels >17.8 ng/mL predict HF re-hospitalization and mortality, and offer a more accurate risk stratification, regardless of age, gender, LV EF, NYHA class, or serum BNP levels (75). Moreover, the predictive value of Gal-3 in patients with HF has been found to be unaffected by the therapeutic strategies used to treat HF or by age (57, 108). However, data comparing Gal-3 predictive value to established biomarkers for HF are conflicting, suggesting that Gal-3 has major prognostic efficacy when used in combination with other HF biomarkers than alone (101).

### GDF-15

Growth differentiation factor-15 (GDF-15), also known as macrophage inhibitory cytokine-1 (MIC-1), is a divergent member of the transforming growth factor (TGF)-β superfamily. In healthy individuals, with the exception of the placenta and prostate, GDF-15 shows low to absent constitutive expression (109). Increased blood levels of GDF-15 are related to stressogenic events, anoxia and acute injury, and are found to be increased in several diseases including inflammation, obesity, cardiovascular disease and cancer (110, 111). In addition, several cell types express GDF-15 under stress conditions, including cardiomyocytes, adipocytes, macrophages, endothelial cells and vascular smooth muscle cells (112). Being an inflammatory and stress-induced cytokine, GDF-15 is also significantly expressed in response to various growth factors and inflammatory proteins, including IL-1ß, TNF-α, IL-2, and macrophage colony-stimulating factor-1, which implies a complex and multidimensional regulation (113). Specifically, GDF-15 appears to play a role in limiting the inflammatory response in the aftermath of tissue damage, reducing leukocyte infiltration and fibrosis (114). Chung et al. found increased expression of GDF-15 in the liver after administration of carbon tetrachloride or alcohol (114). In GDF-15 knockout mice, they also observed an increased degree of liver infiltration by monocytes, CD4+ and CD8+ lymphocytes and macrophages, as well as a lower degree of fibrosis (114). Moreover, the transcription factor p53 binds to the *GDF15* gene promoter region via two distinct binding sites. As a result, GDF-15 expression appears to be linked to the synthesis of p53, which is activated by conditions such as hypoxia, telomere erosion, and oxidative stress (115). This explains why GDF-15 expression rises with aging, a condition known to be linked to several markers of stress and damage, including ROS production, protein glycation, inflammation and hormonal changes.

Elevated serum levels of GDF-15 have been found in several types of cancer (116), also confirmed by biopsy analysis of various tumor tissues (117). Several studies have correlated high levels of GDF-15 with the development of cancer-related anorexia and cachexia (118, 119), as well as worse survival (120). Furthermore, Wallentin et al. in the Uppsala Longitudinal Study of Adult Men (ULSAM) study, identified GDF-15 as an independent predictor of all-cause mortality, cardiovascular and cancer mortality (121). However, the exact role of GDF-15 within tumorigenesis is still unclear, with some evidence supporting its action in promoting malignancy, while others showing its inhibitory effects on cancer. Boyle et al. found that subcutaneous injection of GDF-15-producing metastatic melanoma cells into nude mice resulted in faster tumor development than controls (122). In contrast, some preclinical investigations on transgenic mice have shown that GDF-15 has tumor-suppressing activity: for example, Husaini et al. found that overexpression of this protein reduced tumor mass growth and enhanced survival (123). The conflicting results regarding GDF-15 role in tumorigenesis may be due to the timing of the protein's tumor-promoting or tumor-suppressing effects. It has been hypothesized that GDF-15 primarily performs a tumor-suppressing function in the early stages of tumor development, and then a tumor-promoting function in the later stages (124).

Similarly to cancer, great attention has been paid to the association between elevated serum concentration of GDF-5 and outcomes of various cardiovascular diseases, including atherosclerosis, HF, coronary artery disease (CAD) and ischemic reperfusion injury. By enrolling 3,428 participants in the Framingham Heart Study, Wang et al. demonstrated a strong correlation between high GDF-15 levels, mortality and the development of HF (62). Cotter et al., using data from the RELAX-AHF study, assessed GDF-15 values at admission and at several subsequent time points (125). GDF-15 levels were found to be an independent predictor of short-term cardiovascular mortality and rehospitalization (125). Similarly, in a study on 847 patients with myocardial infarction (MI), using The Proximity Extension Assay proteomics chip (capable to analyze 92 different cardiovascular biomarkers), GDF-15 and TRAIL receptor 2 were identified as the best biomarkers in predicting long-term all-cause mortality (65). Furthermore, synthesis of GDF-15 in the infarcted area of a mouse model of MI was shown to be responsible for reduced leukocyte infiltration, lowering the probability of fatal heart rupture, thus demonstrating the local anti-inflammatory role of GDF-15 (126). GDF-15 has also been identified as a potential biomarker for the risk of cardiovascular events and mortality in patients with ACS and CAD (127). Some preclinical studies have investigated the function of GDF-15 in the pathophysiology of atherosclerosis; however, results have been conflicting, with both a protective and a disease-promoting effect reported in different studies (128). Despite multiple studies strongly linking GDF-15 serum levels with an increased risk of cardiovascular events and death, the specific involvement of the protein in the development of cardiovascular disease is still unknown.

### PlGF and sFlt-1

Vascular endothelial growth factor (VEGF)-A, B, C, D, and E, and placental growth factor (PlGF) are all members of the *VEGF* gene family. The pro-angiogenic action of this glycoproteins is characterized by increased endothelial cell proliferation and survival, as well as improved vascular permeability (129). Furthermore, by serving as a chemoattractant for monocytes and stimulating the production of adhesion molecules on endothelial cells, VEGF has a function in inflammatory responses and ischemic events (130). The pro-angiogenic actions of VEGF are mediated by the VEGFR-2, while the VEGFR-1 (also known as fms-like tyrosine kinase-1, Flt-1) is responsible for sequestering VEGF and functioning as a negative regulator of the neoangiogenic process (131). PlGF, on the other hand, can only bind Flt-1 and its soluble form, sFlt-1, which is the second version of the receptor produced by alternative pre-mRNA splicing. Hypoxia, a stress condition found in both cardiovascular disease and cancer, regulates *FLT1* gene expression and is responsible for the preferential synthesis of sFlt-1. VEGF and PlGF bind to sFlt-1 with a high affinity, allowing it to block their pro-angiogenic activity (131).

PlGF beneficial effects on angiogenesis and cardiac function preservation in post-ischemic myocardium have been investigated in various preclinical studies (132). Furthermore, PlGF appears to be involved in the inflammatory mechanisms supporting atherosclerosis, although data from preclinical studies are conflicting (132). PlGF also seems to play a key role in the cardiorenal connection, possibly as a result of the increased degree of atherosclerosis seen in patients with chronic kidney disease CKD (69). Matsui et al. it have shown that higher serum levels of PlGF are independent predictors of all-cause mortality and cardiovascular events in patients with CKD, with greater strength than traditional risk factors (69); Specifically, patients with plasma PlGF levels of 19.6 pg/mL showed an 8.42-fold increase in cardiovascular mortality and a 3.87-fold increase in all-cause mortality when compared to patients with lower PlGF levels (10.1 pg/mL) (69). A prospective trial of patients with suspected ACS, showed that low serum PlGF and BNP levels were efficient predictors of the risk of MACE, with an incidence at 1 year of <1% (68). Furthermore, an induction of PlGF production within 12 h of MI was observed in patients with ACS without ST-segment elevation, with blood values remaining stable for up to 30 days after the event, implying that PlGF is involved in the healing process of the infarcted area. Interestingly, PlGF serum levels >13.2 ng/L were associated with a greater risk of short-term death (HR 2.28, 95% CI, 1.21–4.7;, *p* = 0.0125), as well as decreased renal function (67). Finally, it was found that PIGF values at baseline were effective prognostic indicators of adverse long-term outcomes in patients with ACS, regardless of platelet activation and myocardial necrosis (10.1016/j.jacc.2005.08.063). As PlGF and sFlt-1 may show independent plasma alterations during ACS, the PlGF/Flt-1 ratio was also investigated (132). Hochholzer et al. found in a sample of patients with symptoms suggestive of acute MI that both biomarkers gave additional prognostic information when compared to established blood biomarker, such as TnT and NT-proBNP (76). Matsumoto et al. also examined the efficacy of the PlGF/sFlt-1 ratio in predicting death from all causes, cardiovascular death, and total cardiovascular events in patients with ACS at baseline (133). The ratio was predictive of an increased risk of adverse events and death from all causes, when compared to the individual biomarkers studied separately. It was also found that a greater PIGF/sFlt-1 ratio value was associated with a higher number of coronary arteries with stenosis at baseline (133).

Through the correct supply of oxygen and nutrients, neovascularization plays a critical role in the growth dynamics of the tumor mass as well as metastases (134). sFlt-1 is expressed in several tumors, including breast cancer, colorectal cancer, and acute myeloid leukemia (AML), and the PIGF/sFlt-1 ratio, as well as sFlt-1, have been linked to the prognosis of a variety of malignancies (135). Furthermore, tumor PIGF synthesis is crucial for keeping the inflammatory response in the TME, and it also appears to induce an immunosuppressive state favorable to tumor growth via NFAT-mediated binding to sFlt-1 (136).

Despite their efficacy in the treatment of a variety of tumor types, the introduction of drugs targeting members of the VEGF family into clinical practice has been linked to cardiotoxicity and the development of a variety of cardiovascular diseases, including hypertension, cardiomyopathy, and deep vein thrombosis (137). These findings emphasize the impact of VEGF in the pathophysiology of cardiovascular disease and cancer, and future studies are needed to clarify the appropriate clinical use of VEGF family biomarkers.

### CHIP

Hematopoiesis is a polyclonal process in which equipotential hematopoietic stem cells differentiate into erythroid, lymphoid, myeloid, or megakaryocytic cells (138). This stem population may develop mutations providing it a proliferative advantage, leading to the formation of clonally expanded stem populations (clonal haematopoiesis), which produce mutant progenies that can be sampled in peripheral blood (139). At the age of 70, more than 10% of people have these clones, which account for about 20% of their peripheral white blood cells on average (71). Most people who are affected by these mutated clones will never develop a hematological malignancy, which is why this condition is called clonal hematopoiesis of undetermined potential (CHIP). It is a pre-malignant state, with rates of progression to hematological malignancy of about 0.5 % per year (71). CHIP is diagnosed when the number of mutant clones in peripheral leukocytes count exceeds 2% (140).

In recent studies, CHIP has been linked to a 2- to 4-fold higher risk of cardiovascular disease (70, 71). Some preclinical studies have also reported an overlap between CHIP and the development of cardiovascular disease: mouse models with mutations in Tet2 (one of the key genes in the pathogenesis of CHIP) showed accelerated formation of atherosclerotic lesions (141). Furthermore, mice knockout for Tet2 showed higher serum levels of inflammatory markers such as C-X-C motif chemokine ligand (CXCL)1, CXCL2, CXCL3, IL-1, and IL-6, implying that Tet2 may have a role in atherogenesis pathogenesis (142).

In a recent retrospective cohort study of 623 patients with AML, 63% of whom carried CHIP-related mutations, Calvillo et al. found an increased prevalence of cardiovascular diseases at baseline (72). Of note, the presence of 1 or more CHIP-related mutations was an independent risk factor for the development of cardiovascular adverse events in patients treated with anthracycline chemotherapy, but not in other patients (72). Furthermore, the timing of the onset of cardiovascular adverse events after a diagnosis of AML was discovered to be a poor prognostic factor, independently related with all-cause mortality (72).

Several concerns remain unanswered in understanding the processes underlying the vascular risk posed by CHIP. More research is needed to understand the genetic and molecular mechanisms behind CHIP, as well as the investigation of environmental risk factors that regulate CHIP, to identify a suitable application for this strong new risk factor. In addition, appropriate screening and treatment strategies for CHIP patients, as well as adjustments in cancer treatment regimens, must be examined.

## Markers of Pro-inflammatory Status

### Pro-inflammatory Cytokines

Aging is associated with the development of a pro-inflammatory status that is characterized by high levels of pro-inflammatory markers in cells and tissues (143). These pro-inflammatory markers include IL-1, IL-6, IL-8, CRP, TGF-β, and TNF-α, among others (143). A systemic inflammatory state may originate from genetic susceptibility, visceral obesity, cellular senescence, impaired recycling and elimination of degraded cellular material, as well intrinsic defects in immune cells and chronic infections (143). It is noteworthy that senescent cells acquire a senescence-associated secretory phenotype that involves the secretion of a wide range of soluble mediators, including IL-1, IL-6, chemokines, growth factors, and metalloproteinases (MMPs) (143, 144).

Despite its fundamental physiological role as a defense mechanism against infections or extraneous agents, when inflammation becomes sustained and prolonged it becomes pathologically detrimental (143). Several studies have indeed shown that inflammation is a risk factor for cardiovascular disease, cancer, CKD, dementia, depression, osteoporosis, sarcopenia, and anemia (143, 145, 146). There is strong evidence suggesting that chronic inflammation is both a risk factor and a pathogenic mechanism in cardiovascular disease; for instance, vascular endothelial cell inflammation participates in the pathogenesis of atherosclerotic plaques, whereas atherosclerosis itself produces antigens that trigger and sustain an inflammatory response (143, 147, 148). In HF, concentrations of several interleukins are increased, including IL-1β, IL-6, IL-8, IL-13, and IL-18, whereas the levels of anti-inflammatory interleukins IL-5, IL-7, or IL-33 are down-regulated (149).

During the last couple of decades, the contribution of inflammation to cancerogenesis has been increasingly recognized. At present, cancer cells are investigated within a network of stromal and inflammatory immune cells that all together form the tumor microenvironment (150). Inflammation drives all stages of cancerogenesis, namely tumor initiation, growth, progression, metastasis, and therapy resistance (150). Furthermore, around 15–20% of all cancers are preceded by infection, chronic inflammation, or autoimmunity; examples of inflammatory pre-cancerous disorders are inflammatory bowel disease, chronic hepatitis, and Helicobacter-induced gastritis (150). Increased IL-6 serum levels seem to be closely associated with cancer patients' clinical condition and to correlate with survival independent of the cancer type (151).

### Myeloperoxidase

Myeloperoxidase (MPO) is a heme-containing peroxidase produced by polymorphonuclear leukocytes (152), which plays an important role in inflammation and microbial killing by neutrophils. MPO has also shown to have pro-atherogenic and pro-oxidant properties, being responsible for lipid peroxidation, nitric oxide (NO) scavenging and NO synthase inhibition (153). MPO may serve as both a marker and mediator of vascular inflammation. In patients with ACS and acute HF, elevated MPO levels importantly predict adverse outcomes and worse prognosis (154, 155).

MPO has also proven to have a promising role in cardioncology (24). In a study by Ky et al., 78 patients with breast cancer undergoing doxorubicin and trastuzumab therapy received a baseline evaluation with 8 different biomarkers: hsTnI, hsCRP, NT-proBNP, GDF-15, MPO, PlGF, sFlt-1, and Gal-3 (156). Among these markers, MPO baseline levels were the only to be significantly associated with cardiotoxicity development (*p* = 0.052). However, interval changes in hsTnI, GDF-15, MPO, sFlt1, and Gal-3 from baseline to 3 months were also associated with subsequent cardiotoxicity (156).

### sST2

Suppression of tumorigenicity 2 (ST2) is a member of the IL-1 receptor superfamily that exists in two main isoforms: a soluble form (referred to as soluble ST2 or sST2) and a membrane-bound receptor form (referred to as ST2 receptor or ST2L) (157). The interaction between IL-33 and ST2L exerts cardioprotective effects in the myocardium by reducing fibrosis, hypertrophy and enhancing survival. The circulating isoform sST2, by sequestering IL-33, abrogates this favorable effect (158). Circulating sST2 is released in response to vascular congestion and inflammatory and pro-fibrotic stimuli, and it serves as a marker of adverse remodeling and fibrosis, cardiac dysfunction, impaired hemodynamics and higher risk of progression. In patients with HF, sST2 is an independent predictor of mortality and HF hospitalization (158).

Serum levels of sST2 and IL-33 were reported to be significantly higher in several different cancer types as compared to healthy controls (159–161). However, their role has not been fully understood yet. Akimoto et al. have shown that sST2 negatively regulates tumor growth and the metastatic spread of CRC through modification of the tumor microenvironment; in particular, sST2 suppresses IL-33-induced angiogenesis, Th1- and Th2-responses, macrophage infiltration and macrophage M2a polarization (162). On the contrary, in other studies sST2 is associated with advanced and metastatic disease in gastric cancer and significantly correlates with the duration of the disease (163).

## Current Recommendations

The 2020 European Society for Medical Oncology (ESMO) consensus recommendations suggest performing a comprehensive baseline cardiovascular risk assessments before starting anticancer therapy (164). This includes baseline measurement of cardiac biomarkers (cardiac troponins and NPs), electrocardiogram, echocardiography (with LV EF and diastolic function evaluation). Biomarkers are useful to detect cardiotoxicity early before changes in LVEF, or clinical signs and symptoms of HF have developed (24). However, biomarkers should not be used in isolation, but rather placed in a comprehensive assessment including imaging, risk factors, clinical symptoms and the cancer specific characteristics (24).

## Therapeutic Perspectives

The assessment of biomarkers in patients with cancer and/or HF provides strong evidence of the physiopathogenetic overlap between these two conditions. The advantage of unveiling the bidirectional relationship between cancer and HF may also rely upon the development of a therapeutical strategy suitable for both (8). To this purpose, inflammation represents an extremely useful target, and anti-inflammatory drugs targeting the IL-1, IL-6, or CRP axis, may lead to improved cardiovascular and oncological outcomes (165). Apart from calorie restriction and physical activity, systemic inflammation can be hindered by small molecules or antibodies interfering with inflammatory mediators or their biological targets (143).

The CANTOS (Canakinumab Anti-Inflammatory Thrombosis Outcome Study) was a randomized, double-blinded, placebo-controlled trial that investigated the use of canakinumab, a monoclonal antibody targeting IL-1β, in over 10.000 high-risk patients with established atherosclerotic disease who had already had a MI) (166). At a median follow-up of 3.7 years, canakinumab led to a significantly lower rate of recurrent cardiovascular events (nonfatal MI, stroke, or cardiovascular death) than placebo (*p* = 0.02074) (166). Strikingly, an exploratory analysis from the CANTOS group revealed that canakinumab can significantly reduce incident lung cancer (HR 0.33; 95% CI, *p* < 0.0001) and lung cancer mortality (HR 0.23; 95% CI, *p* = 0.0002) (167). The analysis also reported that baseline concentrations of CRP (6.0 mg/L vs. 4.2 mg/L; *p* < 0.0001) and IL-6 (3.2 vs. 2.6 ng/L; *p* < 0.0001) were significantly higher among participants subsequently diagnosed with lung cancer than among those not diagnosed with cancer (167).

A meta-analysis of two large prospective cohort studies has also shown that long-term use of aspirin, a nonsteroidal anti-inflammatory drug, was associated with a modest but significantly reduced risk for overall cancer (relative risk [RR], 0.97; 95% CI, 0.94–0.99), which was primarily owing to a lower incidence of gastrointestinal tract cancers (RR, 0.85; 95% CI, 0.80–0.91), especially CRC (RR, 0.81; 95% CI, 0.75–0.88) (168). Similarly, a meta-analysis of four randomized clinical trials, revealed that long-term aspirin intake of at least 75 mg daily reduced long-term incidence (HR 0.76, *p* = 0.02) and mortality (HR 0.65, *p* = 0.005) due to CRC (169).

## Conclusions

Cancer and cardiovascular diseases, more specifically HF, represent some of the most commonly recognized causes of death and comorbidity world-wide. These two entities often share common risk factors and clinical symptoms. While recent findings have highlighted that HF is associated with an increased risk of cancer and cancer-related mortality, heightened in decompensated states (2, 3, 170), common pathophysiological systemic changes (inflammation, metabolism, activation of the neuro-hormonal and immune system, endothelial dysfunction) often subtend both these chronic conditions.

Circulating biomarkers represent a sensitive and specific diagnostic and prognostic tool (as a potential therapeutic target) for evaluation of pre-clinical as well as follow-up of disease condition, potentially aiding in identification of multiple (including cardiovascular) injuries and toxicities.

Although the potentially adverse effects of chemotherapy on the heart are well known (cardiotoxicity), there is limited evidence on the impact of cancer, per sé, on the heart of untreated oncologic patient as well as on the bidirectional relationship between cancer and HF. In pre-clinical and human models, the presence of active cancer has been associated with subclinical metabolic and myocardial cellular damage oncometabolites, (4) and patients with active neoplasms have been shown to have, independently of cardiological comorbidity, increased levels of multiple cardiac biomarkers before chemotherapy, with a demonstrated prognostic role of the same (8).

This milieu might represent, *per sé*, a condition at increased risk of cardiotoxicity, even before chemotherapy. Since oncologists refer patients to cardiological evaluation for risk stratification and monitoring, this evidence might provide further guidance on the management of patients who are candidates to specific treatments: thus far circulating biomarkers represent an easy, bedside and reproducible clinical tool for the entire course of the cardioncological patient, from initial risk stratification (increase of cardiac biomarkers = increased risk for chemotherapy), conventional early detection of cardiotoxicity, short/medium and long term follow-up (survivorship). Finally, circulating biomarkers might aid in the identification of a common pathophysiology between cancer and HF (increase of baseline circulating biomarkers = “cancer cardiomiopathy”), thus prompting the implementation of preventive and treatment strategies on multiple targets (164).

## Author Contributions

MC, GP, IF, SL'A, and DC contributed to the conception of the review, the bibliographic research, and the drafting of the manuscript. AG, AA, GV, CG, VC, CP, CC, AF, and ME contributed to critical revision and editing. MC, GP, and SL'A designated tables and figures. All authors gave final approval and agreed to be accountable for all aspects of work ensuring integrity and accuracy.

## Conflict of Interest

The authors declare that the research was conducted in the absence of any commercial or financial relationships that could be construed as a potential conflict of interest.

## Publisher's Note

All claims expressed in this article are solely those of the authors and do not necessarily represent those of their affiliated organizations, or those of the publisher, the editors and the reviewers. Any product that may be evaluated in this article, or claim that may be made by its manufacturer, is not guaranteed or endorsed by the publisher.

## Abbreviations

AML: acute myeloid leukemia
ANP: atrial natriuretic peptide
BNP: brain-type natriuretic peptide
CA125: carbohydrate antigen 125
CAD: coronary artery disease
CANTOS: canakinumab anti-inflammatory thrombosis Outcome Study
CEA: carcinoembryonic antigen
CHIP: clonal hematopoiesis of undetermined potential
CI: confidence interval
CKD: chronic kidney disease
CRC: colorectal cancer
CRD: carbohydrate recognition domain
CRP: C-reactive protein
cTnI: cardiac troponin I
cTnT: cardiac troponin T
CXCL: C-X-C motif chemokine ligand
ECM: extracellular matrix
EF: ejection fraction
ESMO: european society for medical oncology
Gal-3: galectin-3
GDF-15: growth differentiation factor-15
HF: heart failure
HR: hazard ratio
hs: highly sensitive
ICI: immune checkpoint inhibitor
IL: interleukin
LV: left ventricular
MACE: major adverse cardiovascular events
MI: myocardial infarction
MIC-1: macrophage inhibitory cytokine-1
MPO: myeloperoxidase
MR-proADM: mid-regional pro-adrenomedullin
MR-proANP: mid-regional pro-atrial natriuretic peptide
NO: nitric oxide
NPs: natriuretic peptides
NT-proBNP: N-terminal prohormone of brain natriuretic peptide
PlGF: placental growth factor
RAAS: renin-angiotensin-aldosterone system
RR: relative risk
sFlt-1: soluble fms-like tyrosine kinase-1
ST2: suppression of tumorigenicity 2
TGF-β: transforming growth factor-β
TNF-α: tumor necrosis factor-α
VEGF: vascular endothelial growth factor
VEGFR: vascular endothelial growth factor receptor.
